# TimeTree 5: An Expanded Resource for Species Divergence Times

**DOI:** 10.1093/molbev/msac174

**Published:** 2022-08-06

**Authors:** Sudhir Kumar, Michael Suleski, Jack M Craig, Adrienne E Kasprowicz, Maxwell Sanderford, Michael Li, Glen Stecher, S Blair Hedges

**Affiliations:** Department of Biology, Institute for Genomics and Evolutionary Medicine, Temple University, Philadelphia, PA 19122, USA; Department of Biology, Temple University, Philadelphia, PA 19122, USA; Center for Biodiversity, Temple University, Philadelphia, PA 19122, USA; Center for Excellence in Genomic Medicine Research, King Abdulaziz University, Jeddah 22254, Saudi Arabia; Department of Biology, Institute for Genomics and Evolutionary Medicine, Temple University, Philadelphia, PA 19122, USA; Department of Biology, Institute for Genomics and Evolutionary Medicine, Temple University, Philadelphia, PA 19122, USA; Department of Biology, Temple University, Philadelphia, PA 19122, USA; Center for Biodiversity, Temple University, Philadelphia, PA 19122, USA; Department of Biology, Institute for Genomics and Evolutionary Medicine, Temple University, Philadelphia, PA 19122, USA; Department of Biology, Temple University, Philadelphia, PA 19122, USA; Center for Biodiversity, Temple University, Philadelphia, PA 19122, USA; Department of Biology, Institute for Genomics and Evolutionary Medicine, Temple University, Philadelphia, PA 19122, USA; Department of Biology, Institute for Genomics and Evolutionary Medicine, Temple University, Philadelphia, PA 19122, USA; Department of Biology, Institute for Genomics and Evolutionary Medicine, Temple University, Philadelphia, PA 19122, USA; Department of Biology, Institute for Genomics and Evolutionary Medicine, Temple University, Philadelphia, PA 19122, USA; Department of Biology, Temple University, Philadelphia, PA 19122, USA; Center for Biodiversity, Temple University, Philadelphia, PA 19122, USA

**Keywords:** Evolution, molecular clocks, systematics, timetree

## Abstract

We present the fifth edition of the TimeTree of Life resource (TToL5), a product of the timetree of life project that aims to synthesize published molecular timetrees and make evolutionary knowledge easily accessible to all. Using the TToL5 web portal, users can retrieve published studies and divergence times between species, the timeline of a species’ evolution beginning with the origin of life, and the timetree for a given evolutionary group at the desired taxonomic rank. TToL5 contains divergence time information on 137,306 species, 41% more than the previous edition. The TToL5 web interface is now Americans with Disabilities Act-compliant and mobile-friendly, a result of comprehensive source code refactoring. TToL5 also offers programmatic access to species divergence times and timelines through an application programming interface, which is accessible at timetree.temple.edu/api. TToL5 is publicly available at timetree.org.

## Introduction

The TimeTree of Life (TToL) resource has been delivering scientific knowledge about species divergence times inferred from the analysis of molecular sequences (Hedges et al. 2006; Kumar and Hedges 2011; Kumar et al. 2017). It has assisted many in discovering species divergence times, exploring timetrees, and utilizing them in their research, which is evident from hundreds of annual citations. TToL is also becoming a useful resource for calibrating relaxed molecular clocks in newly studied clades that lack a fossil record (Mello 2018; Mena et al. 2020; Pan and Lin 2020; Duan et al. 2021). Teachers and students also access TToL in the classroom, as do researchers in non-phylogenetic fields and the members of the general public interested in the evolutionary history of life (Babaian 2018; Babaian and Kumar 2020). TToL was featured extensively in the Emmy Award-winning documentary series Rise of Animals hosted by Sir David Attenborough in 2013.

**Fig. 1.**
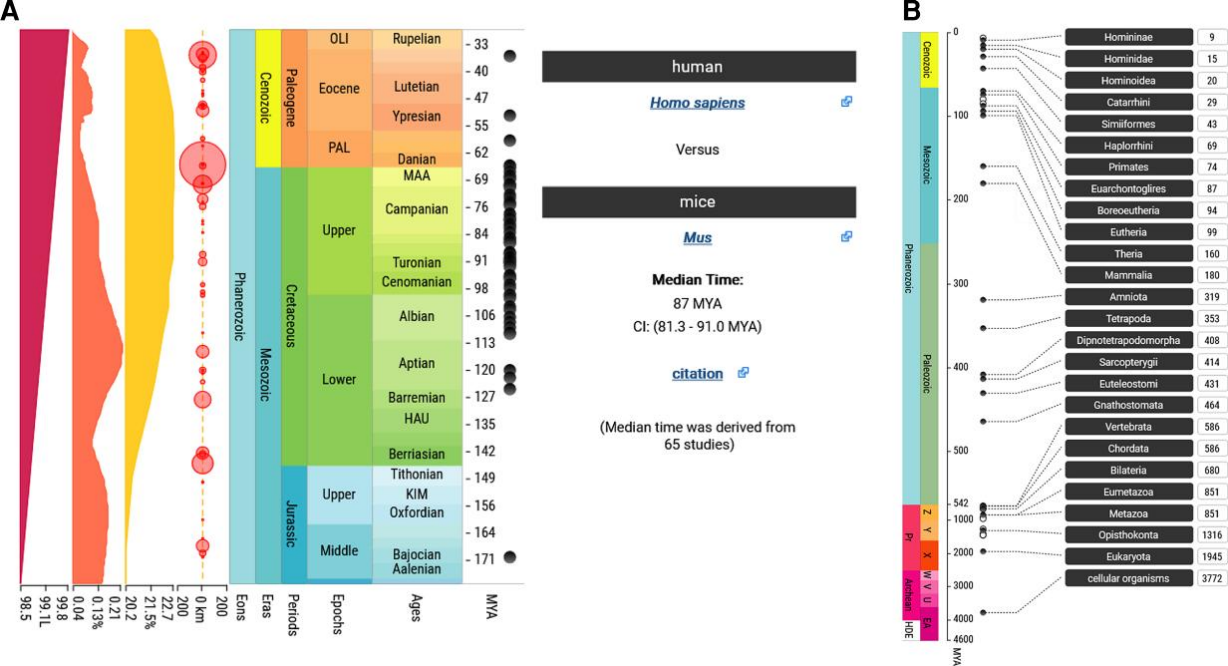
Results produced by TToL5 web portal. (*A*) The divergence time of mice and humans produced by the “Get Divergence Time” search function. The median time and its confidence interval are derived from the TToL5 database. Solar luminosity (furthest left, red), global CO_2_ levels (second from left, orange), O_2_ levels (thirds from left, yellow), and major earth impact events (circles) are also shown and may be toggled by the user. (*B*) The evolutionary timeline of *Homo sapiens* from the origin of all cellular life is produced by the “Get an Evolutionary Timeline” function. Each divergence starting from the root of the timetree of life to Hominidae, the youngest named divergence preceding *Homo sapiens*, is aligned on the evolutionary timescale. Hollow circles indicate divergences without any names in the NCBI database.

Annually, more than 250,000 queries are launched into TToL. The three primary search functions are: “Get Divergence Time,” “Get an Evolutionary Timeline,” and “Build a Timetree.” In brief, the “Get Divergence Time” function takes common (or scientific) names of two taxa. It produces a summary time for their evolutionary divergence along with a list of times reported in individual publications (fig. 1*A*). Divergence times are presented along with earth history and their geological contexts (Kumar et al. 2017). The “Get an Evolutionary Timeline” function produces a series of divergence times between the user-specified species (or taxon) and the origin of cellular life (fig. 1*B*).

**Fig. 2.**
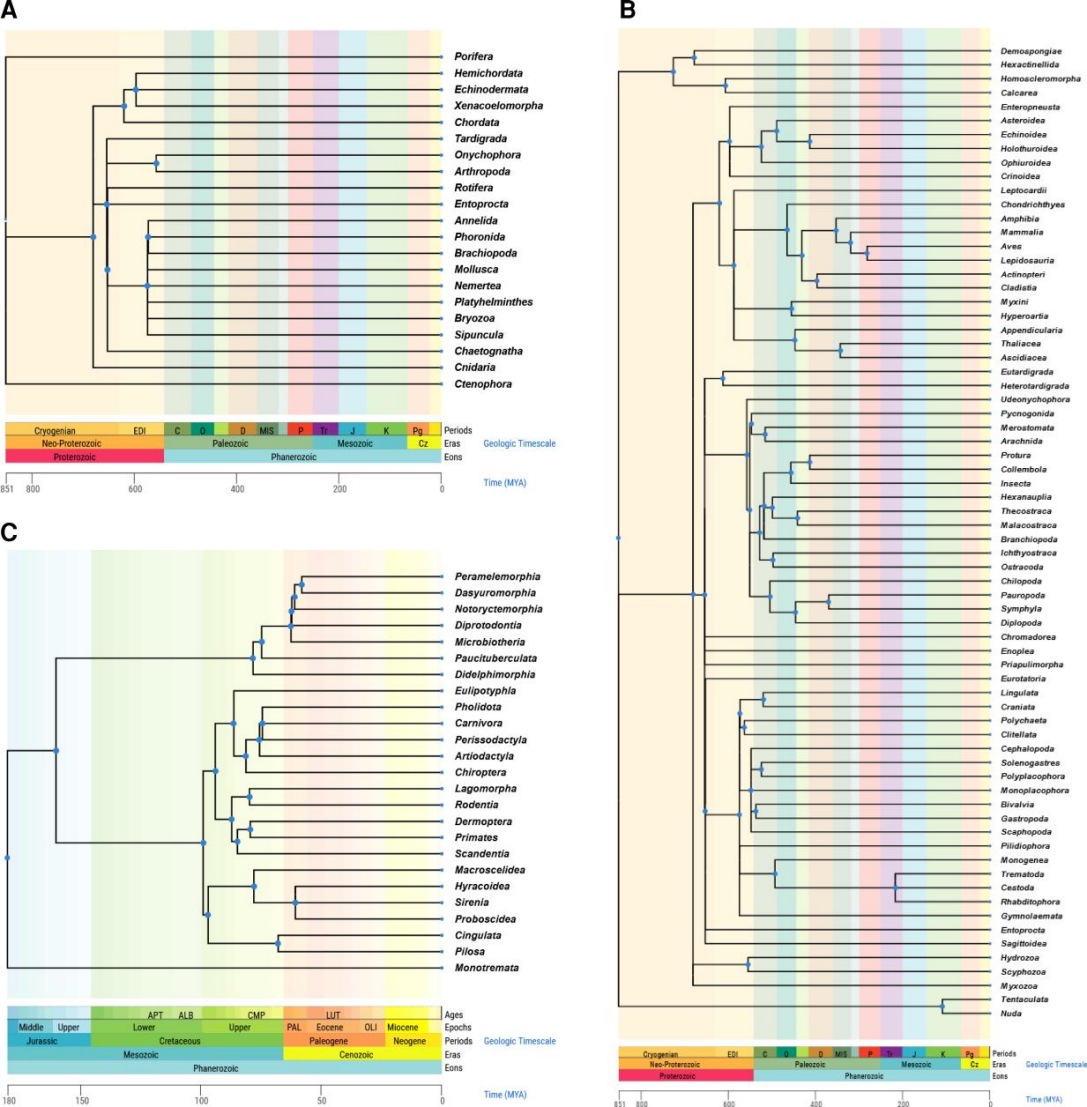
Timetrees produced by the “Build a Timetree” feature in TToL5 to present an overview of the global timetree. Timetrees of (*A*) Animal phyla, (*B*) Animal classes, and (*C*) mammalian orders are shown. Tip labels are removed due to space constraints, and the timescale is shown in millions of years. Blue dots on nodes can be clicked to view the NCBI name, taxonomic rank, median time, and confidence interval around the median. Polytomies reflect phylogenetic uncertainties caused by conflicting resolutions of species relationships and divergence times among published studies included in TToL5. Users can interact with the timetree display in numerous ways (Kumar et al. 2017) and download the resulting timetree in a Newick or graphic format. One can also view individual timetrees and openly download individual published timetrees used in building the global timetree.

The “Build a Timetree” function presents a timetree of taxa of interest extracted from the global timetree connecting species and publication-specific timetrees in the TToL database. One may input a species list or simply give a taxon name to see the clade-specific portion of the global timetree. Options are available to restrict the timetree produced to contain tips at a desired taxonomic level, e.g., species, genus, or family (fig. 2).

Here, we describe advancements in data and technology in the fifth edition of the TToL resource (TToL5).

### Expanded Timetree of Life

**Fig. 3.**
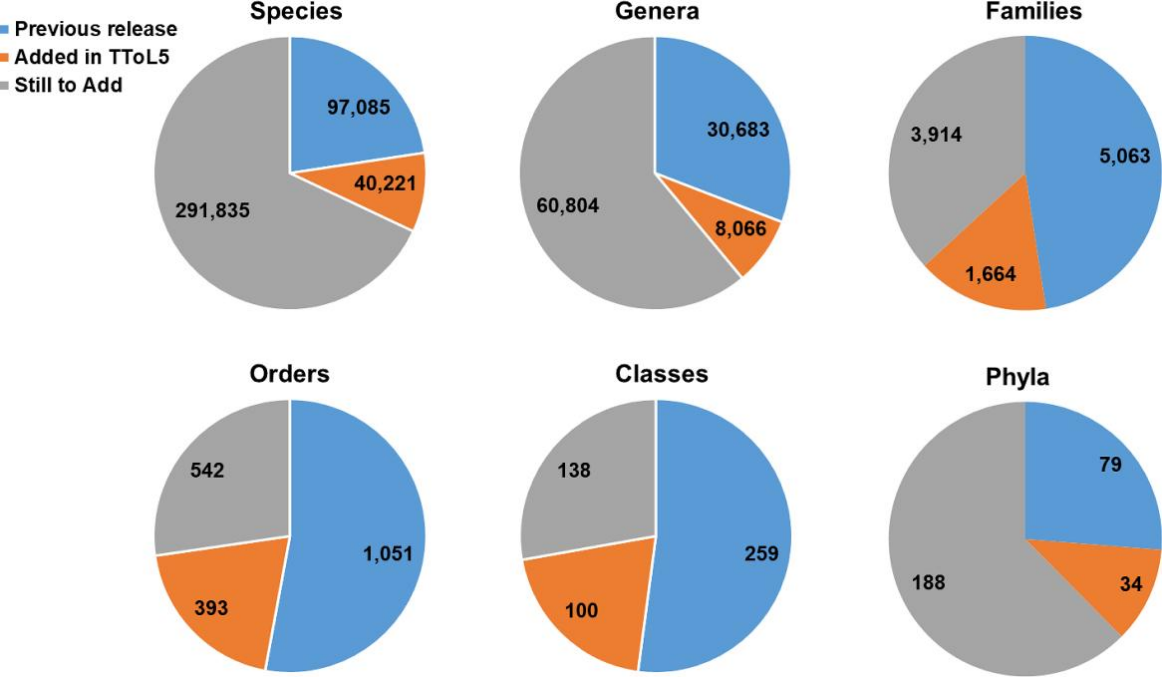
Numbers of species, genera, families, classes, and phyla in TToL5. Orange pies show increased taxonomic representation in this edition compared to the previous edition published in 2017 (blue pies). The grey pies correspond to the number of taxa missing from the global timetree at the given taxonomic level. While the NCBI taxonomy database (Schoch et al. 2020) has over 1.3 million taxa, our count of 429,141 only included species whose names followed binomial nomenclature. This means that species whose whole names included abbreviations such as “sp.” and were marked as environmental samples were excluded. Notably, the number of species with binomial nomenclature fluctuated considerably in NCBI over the last few months (429,141–510,722). We note that 124,654 species names follow the binomial nomenclature in TToL5.

In TToL5, the number of species has increased to 137,306, 41% more than the fourth edition released five years ago (Kumar et al. 2017). The addition of >40,000 species has been achieved through semi-manual curation of many recently published timetrees by the project staff. The increase in species representation has resulted in a 26–43% larger representation of major taxonomic groups (fig. 3). For example, more than 8,000 additional genera are now included, and the number of families has increased by >1,600.

**Fig. 4.**
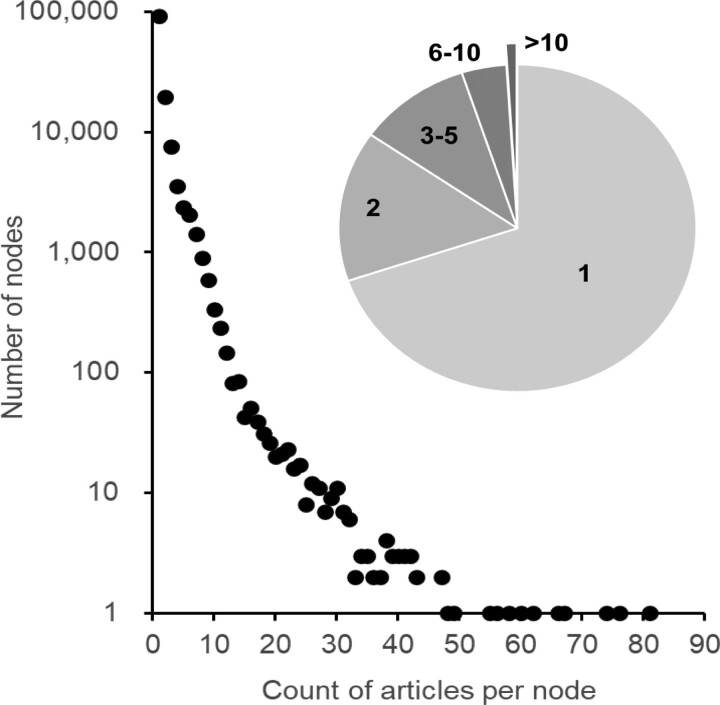
The number of studies informing divergence times for nodes in the TToL5. Divergence times for a vast majority of nodes (*n*, y-axis) are informed by only one study (*s*, x-axis). Their power relationship decays quickly (*n* = 2.17 × 10^5^*s*^–3.015^; *R*^2^ = 0.99), suggesting that only a very small number of node dates are based on many studies, while the vast majority of nodes are only informed by a single article, as shown by the inset pie chart. Species pairs for which the largest number of studies contains the divergence time estimates are the human-chimpanzee comparison (81), mouse-rat and human-old monkey [Old world] comparisons (76), human-mouse comparison (65), and the human-orangutan comparison (62).

TToL5 contains divergence times and timetrees from 4,075 articles published since 1985. They have been synthesized into a global timetree of life following the procedure outlined in Hedges et al. (2015) (see Materials and Methods). The relationship between the number of studies reporting divergence times for clades in TToL5 decays exponentially, such that divergence times for very few nodes have been estimated in more than a few studies (fig. 4). In fact, only a small number of node dates are based on more than 10 studies (fig. 4 inset).

### Technical Advances in TToL5

The TToL5 release includes many technical improvements as well. We have reprogrammed the web pages to become more Americans with Disabilities Act (ADA)-compliant. This effort included rewording the text of download links to make them clearer and more meaningful to screen readers. Internally, the web page source is now restructured to be more appropriate for screen-reading software, making the site navigation easier. For example, headings are added to provide an organized hierarchy of information for users that can be broken into a structure resembling a table of contents. The website is now optimized for navigation using only the keyboard. The white space has been optimized to allow the eyes to relax and digest the content. Foreground and background color contrast have also been increased. Images now provide more meaningful alternatives to text, and hyperlinks are underlined for intuitive navigation. We have also updated web pages to work effectively on mobile devices because researchers and students frequently access their favorite websites on smartphones. These devices have limited screen space. The mobile mode also kicks in when the page size on desktop browsers is too small to accommodate all the information.

In addition, TToL5 now makes available a representational state transfer application programming interface (REST API) for programmatic access to the resource (timetree.temple.edu/api). In the REST API system, one can access information via routes corresponding to some of the major search modes described above. The pairwise API route (/pairwise/) fetches divergence times of two taxa (e.g., species). For example, the human-mouse common ancestor is searched by command/*pairwise/9606/10090.* 9606 and 10090 are NCBI taxonomy identifiers for the human and mouse species. Users can call the “/taxon/human” and “/taxon/Mus + musculus” commands to retrieve these identifiers. One can encode spaces by using a + sign (as ‘/taxon/Mus + musculus’ above) or %20. Note that sometimes a name will resolve to multiple names, requiring the user to choose the desired taxon.

By default, the “/pairwise” query returns a single-row comma-seperated value (CSV)-formatted table of summary information. One can request additional information returned by appending a “/field” flag to the end of the query string, where “field” can be “age’,” “ci,” or “study_count.” The use of a “csv” flag will download a CSV formatted table of divergence times from individual studies, and the “summaryjson” flag will retrieve the summary data as a JSON object.

A generalization of the “/pairwise” function is the “/mrca” route in which one can specify more than two taxa and retrieve the time when their most recent common ancestor (MRCA) existed. It is the crown time of the clade containing the user-specified species in the global timetree. The command is/mrca/id/[NCBI ID list separated by a + ]/field. This query will return results like the pairwise time, with all the same options noted for the “/pairwise” search above.

Finally, the “/timeline” route fetches a list of divergence times of nodes in TToL5 from the user-specified taxon to the common ancestor of all cellular organisms. By default, a CSV formatted table of times is shown. The search string is “/timeline/[Taxon_ID].”

## Conclusions

In summary, the fifth edition of the *TimeTree* resource presents the largest timetree of life ever assembled from published molecular phylogenies. This expansion achieves 20–43% increases in the coverage of major taxonomic groups. TToL5 website is technically advanced, mobile-friendly, ADA compliant, and equipped with an API for programmatic access to the data. We plan to preferentially curate published timetrees covering un- and under-represented taxa in TToL5 with only one or a few studies (figs. 3 and 4).

## Materials and Methods

### Data Collection

Following the approach detailed in Hedges et al. (2015), we identified records of species divergence times, typically in the form of time-calibrated phylogenies (timetrees), by searching and monitoring publication databases such as Google Scholar and PubMed. When timetrees were not distributed with the original publication in the supplementary information, we acquired them from various sources, including databases such as DRYAD, personal repositories maintained by the authors such as GitHub, or via personal communication solicited directly by email or through submissions on www.timetree.org.

The published timetrees were standardized and transformed into computable timetree objects (CTOs). We used in-house software to match the tips of the input timetrees to the NCBI taxonomy database, which frequently required corrections due to misspellings and the use of abbreviations. In-house curating was also necessary to ensure that all timescales were in millions of years. Our curation efforts also ensured that descendant nodes were younger than their ancestors in the individual timetrees added to the database.

### Building the Global Timetree

We used the hierarchical average linking (HAL) approach, introduced in (Hedges et al. 2015), to build a super timetree using CTOs. In HAL, tree topology from NCBI is used as the seed phylogeny, and polytomies are first resolved based on divergence times between pairs of clades; see Hedges et al. (2015) for details. We advanced HAL in some ways. First, when proposing a resolution for a multifurcation, we now prioritize resolutions from timetrees in which the two clades of interest are reciprocally monophyletic. Thus, the divergence time estimates presented are based only on timetrees in which the proposed resolution is supported. Previously, topological uncertainty among the timetrees used for divergence estimates would have caused estimated divergence times biased towards the past. Second, we now rearrange and test local tree partitions iteratively to achieve maximum concordance with the constituent timetrees. This is an improvement over previous partition rearrangements carried out once. Consequently, we expect greater concordance of the super timetree with the constituent timetrees. The resolution of polytomies created many new clades, which were named the same as the name of the NCBI taxon with the polytomy. An asterisk is appended to indicate that the nodes derived their names from the NCBI names.

### Curating the Global Timetree

We first examined the monophyly of genera in TToL5 and found that members of the same genus sometimes occurred in multiple clades. We found these not due to systematic error but rather to understudied clades needing taxonomic revision or outdated terminology in the backbone. Thus, while the positions of individual species often matched the topology found in recent publications, mismatches in the terminology led to the appearance of polyphyletic genera. We also found that the inclusion of some studies caused large time swings between the new and the previous TToL editions. Typically, this occurred in cases where the study’s primary objective was not systematic, e.g. (Kim et al. 2013), which focused on the structural evolution of the chloroplast, constructed a data-poor four-taxon timetree with dates discordant with the rest of the literature. Similarly, Picciani et al. (2018) focused on the evolution and morphology of cnidarian eyes rather than timetree estimation. In all, we found 13 studies containing multiple node times that differed from those present in other studies by more than 5-fold. They were excluded from global timetree calculations. Also, we found several very large-scale phylogenies, e.g. (Tonini et al. 2016; Rabosky et al. 2018), to consistently report times older or younger than those reported in other studies. Phylogeny time imputations and other factors could cause this, but investigating it more thoroughly was outside this project’s scope, so we retained these timetrees in our database.

### Estimation of MRCA

In TToL5, we require reciprocal monophyly of two offspring clades in individual studies. This means that when a pair of taxa is given, their MRCA and its two relevant descendant clades are first determined, and then the study times are extracted if these clades are reciprocally monophyletic. This reduces the number of studies available for some dating a node in TToL5 compared to the previous version. We use the median for aggregating times across studies, which is intended to minimize the impact of outliers. When more than two studies dated a divergence, the confidence interval around the median is presented around the median. Otherwise, a range of times is given for nodes to which times from exactly two studies are mapped. In addition to median times and their confidence intervals, we present “adjusted times” when a node is older than its parent in the global timetree reconstructed from individual timetrees. It will likely result from large uncertainty associated with individual time estimates, differences in calibrations and other assumptions used in different studies, and the presence of only one or a few studies that have dated a species divergence. In brief, when the divergence time for node *b*, *t*(*b*) is older than its parent node *a*, we scan divergence times of all the direct descendants of *b* to find the oldest child of *b* (node *c*) such that *t*(*c*) > *t*(*b*). If there are no such situations, then we set *t*(*c*) = *t*(*b*). Then, the adjusted node times for *a*, *b*, and *c* are set to be the average of *t*(*a*) and *t*(*c*). This adjustment process is carried out repeatedly for the parent node *a* until the adjusted time t*(*a*) ≥ adjusted time t*(b). Then, the process is repeated for all the direct descendants of *b*. This adjustment process can result in the creation of multifurcations. In the pairwise and timetree displays, we show adjusted times if it is not the same as the median time.

## Data Availability

All standardized timetrees can be downloaded using the TToL5 GUI for use in research and teaching (see timetree.org for details on usage). Curated individual timetrees from published articles can be downloaded from the TToL GUI via the “studies” tab and other tabular displays. The collection of all individual timetrees can be requested by emailing info@timetree.org for use in individual research and methods development.
